# A Perspective on the Application of Pro-/Synbiotics in Clinical Practice

**DOI:** 10.3389/fmicb.2017.00866

**Published:** 2017-05-23

**Authors:** Xiang-Dong Wu, Yu Chen, Wei Huang

**Affiliations:** ^1^Department of Orthopaedic Surgery, The First Affiliated Hospital of Chongqing Medical UniversityChongqing, China; ^2^Evidence-Based Perioperative Medicine 07 Collaboration GroupHong Kong, Hong Kong

**Keywords:** probiotics, prebiotics, synbiotics, microbiota, clinical practice

Our previous meta-analysis (Wu et al., 2017) evaluated broadly the available evidence and confirmed that pro-/synbiotics supplementation is effective in preventing or controlling the incidence of surgical site infections (SSIs) after a surgical procedure, yet subgroup analyses indicated the primary outcome was influenced by various factors (Figure 1), which might affect the robustness of the conclusion and further confuse the clinical practice. Lack of proper application of pro-/synbiotics is the primary cause of ineffective management. Thus, several issues around the practicalities of pro-/synbiotics should be taken into account.

**Figure 1:**
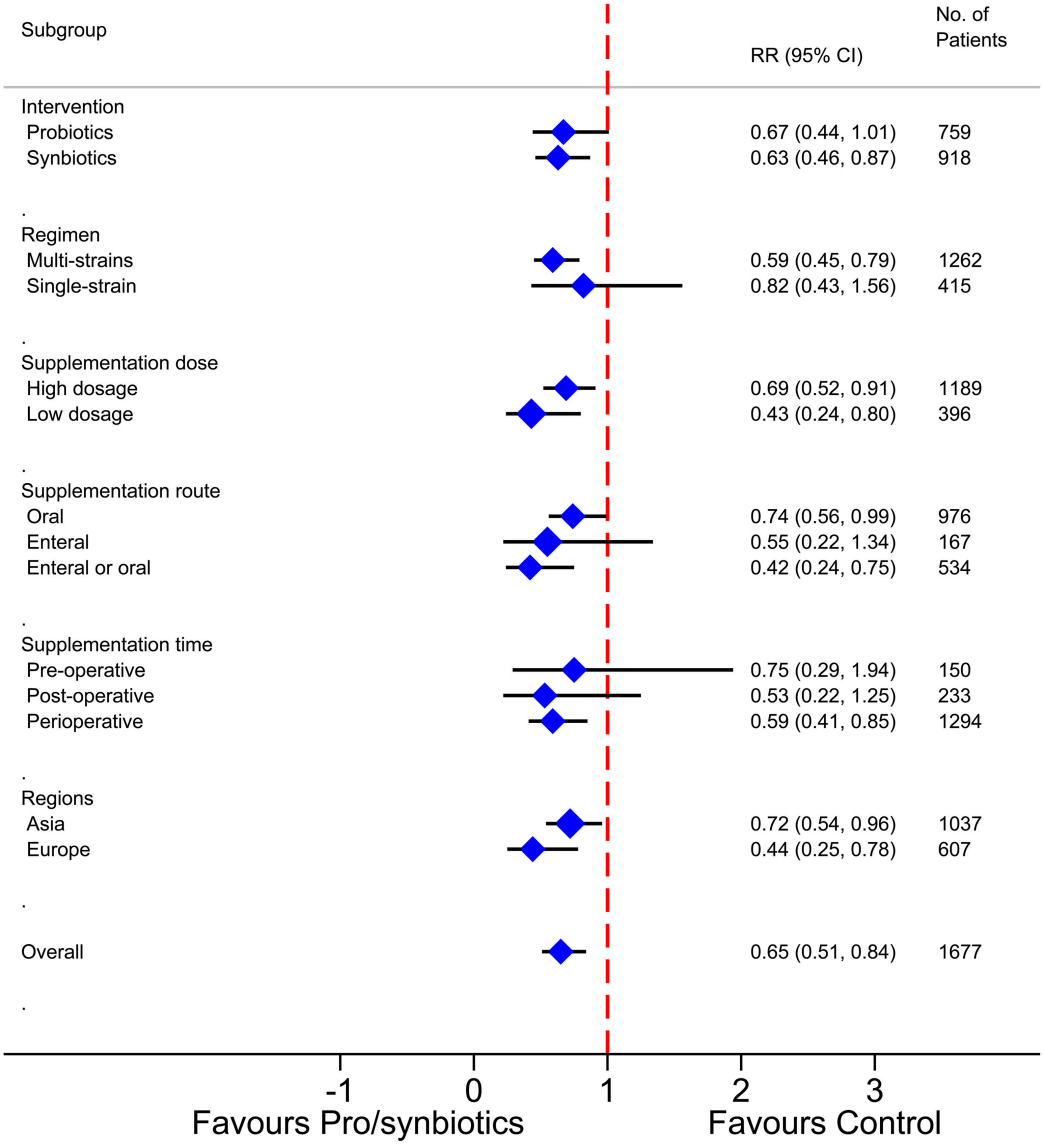
**Subgroup analyses of perioperative pro-/synbiotics for surgical site infections in surgical patients**.

## What are the best regimens or constitutions?

Theoretically, prebiotics could reach the colon intact and selectively stimulate the growth and activities of probiotics, and synbiotics combining probiotics with prebiotics should work better than probiotics alone (Gibson and Roberfroid, 1995). However, whether synbiotics therapy provides greater benefit than either the probiotics or prebiotics on its own still need further directly head-to-head comparison (Tang and Lodge, 2016).

We also detected multi-strains are more effective than single-strain, this might attribute to a synergic effect (Timmerman et al., 2004). But the subsequent issue is all pro-/synbiotics often get lumped together, which consist of many different species and concentrations vary wildly between products. As such lumping studies together unselectively is prone to lead to conflicting results. Therefore, further trials applying slightly more selective probiotics in homogenous population would conduce to show a bit more consistent results.

Previous studies have established that individual probiotics can have distinct strain-specific effects (Luyer et al., 2005; Kekkonen et al., 2008; Tang et al., 2010; Frei et al., 2015). Similarly, different prebiotic oligosaccharides have different microbiota-modifying and immunomodulatory properties (Lee and Salminen, 2009). Hence, investigation of individual probiotics and prebiotics effects is conducive to designing and using combined regimens for specific clinical conditions. Another weak point of many studies done in the field of pro-/synbiotics is the carrier, which may partially influence the effectiveness (Moradi et al., 2013; Mohammadmoradi et al., 2015).

One more major problem is the usage of wrong strains, some reported probiotics bacteria are even among known pathogens (Boyle et al., 2006; Hempel et al., 2011). Microbiologists have explained that the behavior of a microbe depends on several factors, we are ignoring the warnings (Sanders et al., 2010; Pirofski and Casadevall, 2012; Didari et al., 2014; Doron and Snydman, 2015). *In vitro* and *in vivo* tests on a range of probiotics cultures for their ability to inhibit a panel of pathogens are desperately needed to establish an effective routine (Papadimitriou et al., 2015). It should be further emphasized that strains used in multi-strains and multi-species of probiotics and prebiotics should be compatible or, preferably, synergistic (Timmerman et al., 2004). Overview, emerging reports manifest that we should choose the right strains alongside with proper prebiotics and adequate carrier to reach a synergic effect. But the best regimens or constitutions still need copious further studies.

## How to use the regimen?

It should be a state-of-the-art technique to use pro-/synbiotics regimen to modulate the gastrointestinal microbiota. But the key question is we know very little about how to use the regimen appropriately.

Subgroup analysis favors high does rather than low does of pro-/synbiotics. It may be mainly due to that the human microbiota contains as many as 10^14^ bacterial cells, and majority reside in our colon where densities approach 10^11^ ~10^12^ cells/g, which is the highest record for any microbial habitat bacterial cells (Savage, 1977; Whitman et al., 1998; Ley et al., 2006). Thus only high does more than 10^10^ CFUs/day can reach and colonize the gut to further inducing changes in the colorectal microbiota and stabilize microbial communities (Hemarajata and Versalovic, 2013). And studies with inadequate dose of strains didn't experience any improvements. Nevertheless, one dose level cannot be assumed to be always effective for all strains (Sanders, 2008).

The mode of administration is rather important, because pro-/synbiotics are fragile and can be killed easily by heat or stomach acid (Alvarez-Calatayud and Margolles, 2016). This explains why enteral/oral route is more effective than oral and enteral. It is still difficult to measure potential probiotics that survival and colonization to the gut wall. The impact of product format on pro-/synbiotics function has yet to be explored in depth.

The timing for the colonization and proliferation of the gut by the probiotics is also important. We found peri-operative administration is more effective than pre-operative or post-operative administration, this may mainly due to the slow rate of cell division (Lee et al., 2004). Thus longer administration would accelerate the accumulation of microbes, and altered which into a healthy microbiota.

One more interesting issue is the regional difference. Subgroup analysis indicated pro-/synbiotics are more effective in Europe than Asia. We all know that the food habit and environmental condition change the microflora, which would induce intestinal microbiota difference in distinct areas (Marathe et al., 2012). Therefore, we should also take regional specificity into consideration when developing the best regimens.

The statement that pro-/synbiotics “improve the balance of microflora” is often declared by the producers. Although our understanding of the composition and functions of the gut microbiota has increased exponentially during the past decade (Arumugam et al., 2011), we still don't know exactly what roles most of the intestinal bacteria are playing, and how they are interacting with each other and the hosts. Gastrointestinal tract remains a challenging environment to explore, sample, and to describe (Marchesi et al., 2016). Dose colonize, proliferate, and alter the population corresponding to an “improved balance”? Without more knowledge of the larger percentage of unknown microbiota, we cannot learn about whether or not the gut microbiota is a potential therapeutic target which we can modulate in order to treat or prevent specific diseases (Marchesi et al., 2016).

Although there is evidence to support pro-/synbiotics use in reducing SSIs for patients undergoing a surgical procedure, strong scientific evidence to support specific uses of pro-/synbiotics for most health conditions is lacking. Therefore, the U.S. Food and Drug Administration (FDA) has not approved any pro-/synbiotics for preventing or treating any health problem. Absence of a legal definition allows many pro-/synbiotics are sold as dietary supplements on various levels of quality. The cost of these products can be substantial and may not be covered under patients' health care plan (Matarese et al., 2003; Visich, 2010).

## Call for further studies

Considering the current encouraging evidence and challenges, more research in humans to further document the health benefits of pro-/synbiotics as adjunct therapy are needed (Schaeffer, 2017). First, well-designed and properly powered trials with appropriately chosen of strains should be performed, current literature reported conflicting observations may partly be due to poor study design and poor choice of strain (Marchesi et al., 2016). Next, further studies are needed to explore the strain specificity, does specificity, strain combinations characterized for the specific health target, and ultimately achieve using predefined administration mode of specific pro-/synbiotics regimen for definite disease in certain population or region. Third, the effect of product format on pro-/synbiotics function also needs to be explored in depth. Apart from viable bacterial density, other factors like pro-/synbiotics growth during manufacture, enteric coating, preservation technology, metabolic state, and combination with other functional ingredients, may also play a role in the effectiveness of a product (Sanders, 2008). Finally, more research is warranted to understand the human microbiota, there is also a persistent lack of understanding as to the very nature of pro-/synbiotics. We could only speculate that pro-/synbiotics may actually facilitate a return to normal balance status after a perturbation of the microbiota (e.g., because of the use of antibiotics, traditional mechanical bowel preparation or surgical stress) or may reduce the degree of change invoked by such challenges (Sanders, 2008). Therefore, better understanding the mechanisms of the pro-/synbiotics interactions with microbiota would contribute to elucidating how these benefits are achieved, as well as developing novel therapeutics and strategies to modulate the microbiota (Ford et al., 2014).

## Limitations

Our opinion has limitations. First, the above mentioned clinical application perspective was based on the results of six subgroup analyses, and the results of the subgroup analyses should not be interpreted as definitive conclusions since they are observational by nature and are not based on randomized comparisons (Sun et al., 2010). Furthermore, the results of test of interaction suggest no significant differences between subgroups; all subgroup analyses were not specified a priori but *post hoc* analyses, the numbers of studies in the “significant subgroups” are occasionally small, and it is more likely to overestimated the intervention effect compared with larger sample size (Kjaergard et al., 2001; Sterne and Egger, 2001). Therefore, these subgroup analyses results should be interpreted with caution as we might not have had enough power to detect a difference. Next, the included studies were methodologically and biologically heterogeneous, which mainly reflected in the huge variability in clinical settings, pro-/synbiotics strains, routine supplementation does, administration route, control intervention, and stringency of trial execution. These varieties induced equivocal results and further limited the validity and generalizability of our findings. However, our perspective still pointed out the shortcomings of the current research field, and strengthened the keystone for further clinical practice that were worth investigating or revisiting. Lastly, further trials should pay additional concern to the conflicts of interest. Few included studies mentioned industry funding, and small prospective studies sponsored by the pro-/synbiotics industry are likely to be biased (Bekelman et al., 2003; Bero, 2013).

Currently, it is hard to give an exhaustive advice or elaborate guidance on pro-/synbiotics application in clinical practice regarding preliminary findings. To some extent, synbiotics combined multiple-strains probiotics with prebiotics, administrated perioperatively at a high dose should be more effective. To play the greatest degree of pro-/synbiotics in clinic, considerable amount of *in vitro* work are warrant, and intensive *in vivo* exploration followed by randomized, double blind, placebo controlled clinical trials need to be performed.

## Author contributions

XW: Contributed substantially to conception and design, acquisition of data, analysis and interpretation of data; drafted the article; gave final approval of the version to be published; agreed to act as guarantor of the work. YC: Contributed substantially to acquisition of data, analysis and interpretation of data; drafted the article; gave final approval of the version to be published; agreed to act as guarantor of the work. WH: Contributed substantially to conception and design, acquisition of data, analysis and interpretation of data; revised it critically for important intellectual content; gave final approval of the version to be published; agreed to act as guarantor of the work.

### Conflict of interest statement

The authors declare that the research was conducted in the absence of any commercial or financial relationships that could be construed as a potential conflict of interest. The reviewer GSu and handling Editor declared their shared affiliation, and the handling Editor states that the process nevertheless met the standards of a fair and objective review.
